# The potential role of artificial intelligence in the clinical management of Hansen’s disease (leprosy)

**DOI:** 10.3389/fmed.2024.1338598

**Published:** 2024-03-08

**Authors:** Patrícia D. Deps, Rie Yotsu, Brunna C. R. S. Furriel, Bruno D. de Oliveira, Sergio L. de Lima, Rafael M. Loureiro

**Affiliations:** ^1^Department of Social Medicine, Health Sciences Center, Federal University of Espirito Santo, Vitoria, Brazil; ^2^Department of Radiology, Hospital Israelita Albert Einstein, São Paulo, Brazil; ^3^Department of Tropical Medicine, Tulane University School of Public Health and Tropical Medicine, New Orleans, LA, United States

**Keywords:** artificial intelligence, leprosy, Hansen’s disease, digital health, skin neglected tropical diseases

## Abstract

Missed and delayed diagnoses of Hansen’s disease (HD) are making the battle against it even more complex, increasing its transmission and significantly impacting those affected and their families. This strains public health systems and raises the risk of lifelong impairments and disabilities. Worryingly, the three countries most affected by HD witnessed a growth in new cases in 2022, jeopardizing the World Health Organization’s targets to interrupt transmission. Artificial intelligence (AI) can help address these challenges by offering the potential for rapid case detection, customized treatment, and solutions for accessibility challenges—especially in regions with a shortage of trained healthcare professionals. This perspective article explores how AI can significantly impact the clinical management of HD, focusing on therapeutic strategies. AI can help classify cases, ensure multidrug therapy compliance, monitor geographical treatment coverage, and detect adverse drug reactions and antimicrobial resistance. In addition, AI can assist in the early detection of nerve damage, which aids in disability prevention and planning rehabilitation. Incorporating AI into mental health counseling is also a promising contribution to combating the stigma associated with HD. By revolutionizing therapeutic approaches, AI offers a holistic solution to reduce the burden of HD and improve patient outcomes.

## 1 Introduction

Hansen’s disease (HD), commonly known as leprosy, is a contagious and chronic infectious disease. It experienced a resurgence in new cases after a reduction during the COVID-19 pandemic in 2020 (1, 2). Reflecting this shift, the number of countries reporting HD information increased from 143 in 2021 to 182 in 2022. Globally, 174,087 new cases were reported in 2022, representing a 23.8% increase compared to the 140,594 cases reported in 2021. The rise in newly detected cases in 16 of the 23 global priority countries in 2022 was attributed to improved leprosy services following the pandemic. While the overall increase in all priority countries was 25% compared to 2021, the growth varied from 3.4% in Micronesia to 107.4% in Myanmar (3).

HD is caused by *Mycobacterium leprae*; in some regions, *Mycobacterium lepromatosis* is also involved (4). The incubation period is long (average of 5 or more years). The bacillus is likely transmitted through droplets from the nose and mouth during prolonged close contact with untreated patients (5).

HD is considered a clinical and immunological spectral disorder that primarily affects the skin and peripheral nerves. Nerve damage is not uncommon and affects sensory and motor functions in areas such as the eyes, hands, and feet. This condition can be diagnosed by identifying well-established clinical indicators and symptoms, including a range of skin lesions, and detecting peripheral nerve damage, such as sensory loss and (palpable) thickened peripheral nerves. Diagnosis confirmation can be achieved by detecting acid-fast bacilli in slit skin smears (SSS) or by specific findings in samples obtained from skin and nerve biopsies. Moreover, laboratory techniques such as molecular methods for detecting the *M. leprae*-specific repetitive element RLEP in SSS and skin biopsy, along with anti-phenolic glycolipid I (PGL-I) serology titers, play a supporting role in clarifying cases, monitoring patients and household contacts, and assessing subclinical infections within the community (5, 6).

This article discusses how artificial intelligence (AI) can be instrumental in managing HD. AI’s applications include classifying cases, selecting treatment regimens, assessing therapy compliance, and monitoring local and national treatment coverage. It can also assist in detecting adverse drug reactions and antimicrobial resistance. Moreover, AI may enable the early detection of nerve damage, thereby preventing disabilities and aiding in planning rehabilitation strategies. AI can also contribute to mental health management by counseling affected individuals and their family members, thus combating stigma directly and indirectly.

## 2 World health organization’s initiatives

The World Health Organization has set a target of eliminating (or interrupting) transmission in 120 countries by 2030 (7). The specific target for HD in 2030 is defined as “an epidemiological state in a leprosy-endemic country or area where there is no more local transmission of *M. leprae*,” demonstrated by zero new autochthonous cases in children under 15 years of age for at least 5 years (8). Of the 10 challenges that need to be addressed within this timeframe to achieve this goal, two are related to delays in diagnosis and the lack of HD specialists. In addition, this disease is strongly associated with stigmatization. Discrimination against people affected by HD is an obstacle to achieving the elimination target. Therefore, combating stigma to ensure that human rights are respected is one of the four strategic pillars to achieve the goals of the “Zero Leprosy Roadmaps” in all endemic countries (7).

## 3 Challenges in the diagnosis and treatment of Hansen’s disease

### 3.1 Late diagnosis

Delayed (or missed) diagnosis of HD can lead to serious health problems, including disability and social exclusion. HD is usually diagnosed based on clinical signs and medical history. However, untrained healthcare professionals, limited access to medical facilities (especially in low- and middle-income countries), and the stigma associated with HD often prevent timely diagnosis and treatment. Therefore, this delay represents an obstacle to the effective management of HD (6).

### 3.2 Adverse drug reactions due to multidrug therapy (MDT)

MDT is the standard treatment for HD and typically consists of a combination of drugs, including dapsone, rifampicin, and clofazimine. While MDT is highly effective in curing HD, it can also be associated with adverse drug reactions. An analysis of 194 patient records revealed that 88 patients (45%) had side effects linked to MDT, leading to the discontinuation of the suspected drug in 47 of 88 cases (24% of patients) (9). These adverse drug reactions vary in severity and occurrence among individuals, and healthcare providers monitor patients closely during MDT to manage any adverse effects (6).

MDT has been a cornerstone in the global effort to combat HD and reduce its prevalence. However, emerging concerns about drug resistance highlight the importance of continued research and personalized treatment approaches to ensure the continued effectiveness of HD treatment. Considering the patient’s specific *Mycobacterium leprae* strain and their clinical and genetic factors, this approach is critical in ensuring that HD remains a curable and manageable disease despite evolving drug resistance challenges (5).

### 3.3 Underdiagnosis

There is a suspicion of underdiagnosis of HD cases in endemic countries. The discrepancy between expected and observed cases amounted to around 2.6 million from 2000 to 2012 and was projected to exceed 4 million by 2020 (10). The COVID-19 pandemic caused a 40% drop in new HD cases in Brazil in 2020 (1). This highlights a substantial pool of undiagnosed individuals, contributing to ongoing transmission and burden for affected individuals and public health systems.

## 4 Potential role of AI in HD programs

**Figure 1:**
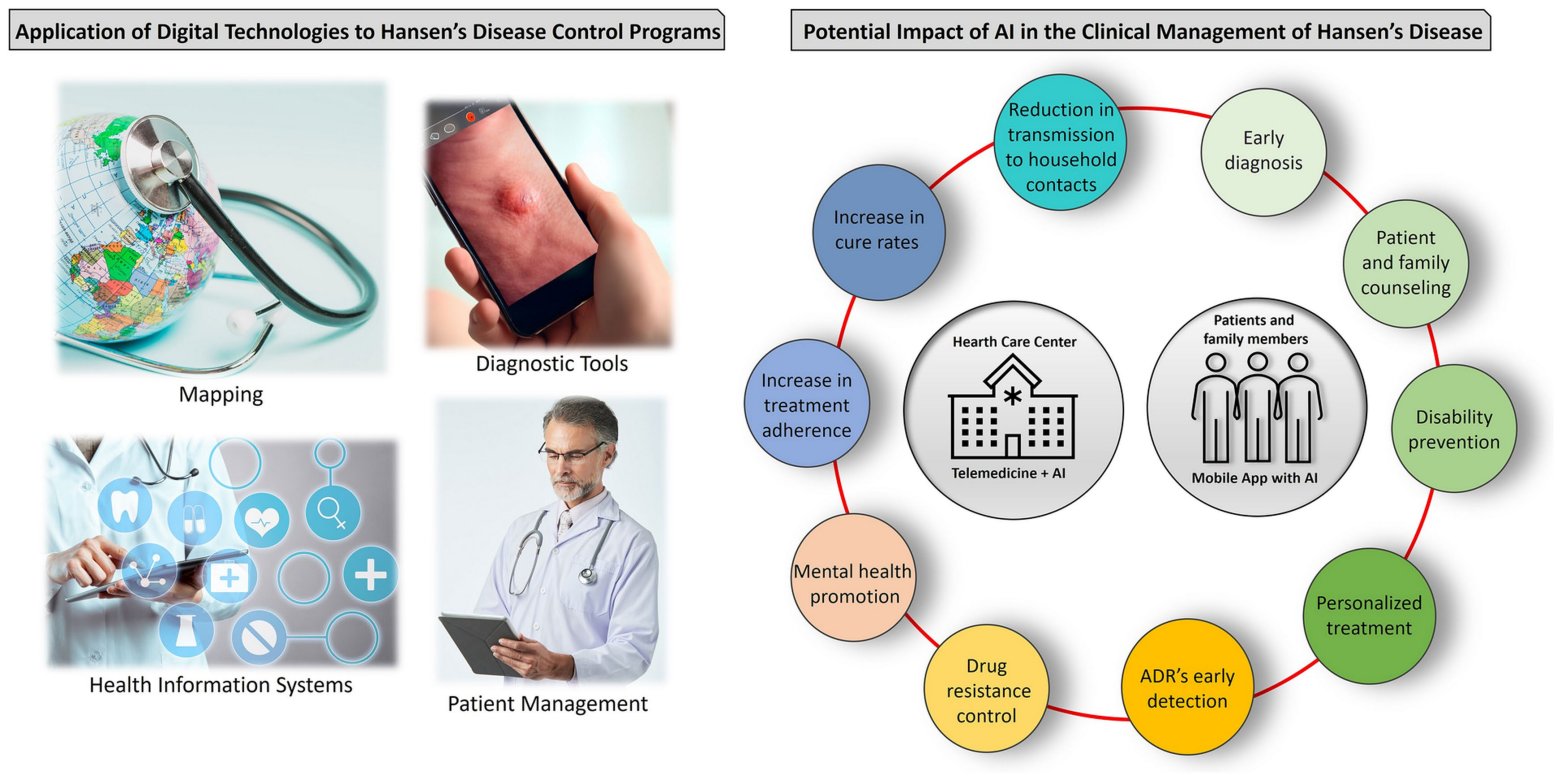
Integration of digital technologies and artificial intelligence (AI) in Hansen’s disease (HD) management. The application of digital technologies involves digital mapping, accurate diagnosis through imaging, efficient data management, and communication among healthcare providers. AI predicts treatment outcomes, enhances cure rates, and ensures treatment adherence. It also detects drug resistance and adverse reactions early, supports early diagnosis, offers mental health counseling, and tailors patient-specific treatment plans. AI-integrated mobile apps provide educational resources, improving remote patient care and disease management. ADR, adverse drug reactions.

The rapid progress of digital technologies offers great potential for transforming the approach to HD programs. At the International Federation of Anti-Leprosy Associations (ILEP) conference in 2020, various participants explored the application of new digital technologies to enhance HD services. ILEP members are adopting state-of-the-art technologies in mapping, diagnostics, health information systems, and patient management (11). This underscores the importance of staying updated on emerging technological trends to improve HD programs and ultimately enhance the well-being of individuals affected by the disease. Figure 1 illustrates the integration of digital technologies and AI in HD management.

## 5 Role of AI in diagnosis of HD

The comprehensive strategy outlines decisive steps to combat HD worldwide, especially in regions with a shortage of specialized physicians. First and foremost, AI could be key in screening and diagnosing new HD cases, thus bridging this “expertise gap.” This includes implementing initiatives to identify cases and contacts in regions with elevated HD detection rates, often referred to as HD hotspots, with prior education campaigns at community level. In addition, the evaluation of diagnostic tools such as antibody detection and molecular techniques is particularly valuable in identifying multibacillary cases, which account for approximately 69% of new HD cases globally (12).

Machine learning, a branch of AI, enables computers to learn from data and make decisions without explicit instructions. It is widely used in areas such as image recognition and speech processing. In healthcare, machine learning models can be trained to recognize skin lesions by analyzing images to identify disease patterns. Machine learning algorithms can also analyze patient data for diagnosis and risk prediction. These technologies, when validated and available in mobile apps, could significantly improve timely HD diagnosis (11, 13–15).

AI algorithms to diagnose HD will initially classify people as suspected cases. However, a “human” doctor will make the final confirmation. AI can be a valuable tool for early detection and identification, helping medical experts make more accurate and efficient diagnoses. Finally, the availability of algorithms capable of diagnosing HD also signifies that an increasing number of new cases can be detected.

AI effectively recognizes the typical clinical presentations of HD (13–15), which experienced dermatologists or well-trained physicians can readily identify. However, laboratory tests can be invaluable for atypical clinical manifestations, asymptomatic individuals, or subclinical cases of HD, including SSS, skin or nerve biopsy, RLEP quantitative polymerase chain reaction (qPCR), and anti-PGL-I serology (5).

## 6 Treatment personalization with AI

HD manifests differently among patients, requiring tailored treatment approaches for better outcomes and fewer complications. AI-driven personalized HD treatment starts with the comprehensive collection and combination of various patient data. This includes medical history, genetic information, clinical evaluations, past treatments, and socioeconomic background. The remarkable ability of AI to analyze this abundance of information offers a comprehensive view of the patient’s situation, leading to more precise treatment decisions (16).

AI can contribute significantly to personalized HD treatment through two key aspects: risk stratification and prediction of individual response to therapy. By analyzing the collected data, AI algorithms can classify patients into risk groups based on factors such as HD type, bacterial load, and comorbidities. This risk stratification allows healthcare providers to allocate resources more effectively and prioritize high-risk patients who require closer monitoring and specialized care. In addition, based on a large dataset of treatment outcomes, AI can identify patterns and factors that influence the effectiveness of specific treatments for individual patients. Equipped with this predictive capability, healthcare providers can make informed decisions about drug selection and dosing, minimizing the risk of adverse effects, drug resistance, and treatment failure (17, 18).

The role of AI can go beyond the prescription phase and actively participate in the patient’s treatment journey. AI-powered applications can provide real-time reminders and support and ensure that patients adhere to their treatment plans. This technology improves not only treatment outcomes but also the patient experience by providing personalized guidance and encouragement.

## 7 AI revolutionizing stigma reduction and early diagnosis

Stigma often acts as a significant barrier to individuals seeking timely medical care (19).

AI-powered chatbots and symptom-checking applications offer individuals a discreet way to assess their symptoms and provide benefits such as anonymous assessment, education, and mental health support. These tools allow people to seek information without fear of social stigma or judgment. They ensure confidentiality and personalization of information for greater engagement and address concerns in a non-judgmental and empathetic way (20).

AI-driven chatbots and educational platforms can be employed to develop and disseminate educational materials and campaigns, dispel myths, and reduce fears. Mental health chatbots help those facing emotional issues due to stigma by offering coping strategies, connecting people with support groups, or recommending professional counseling when needed (21). Also, these chatbots can offer AI-driven translation services to bridge language barriers and make accurate information accessible to individuals regardless of their language.

AI tools can monitor social media and online platforms for discussions related to HD. Sentiment analysis can identify negative or stigmatizing discussions and enable organizations to intervene with accurate information (22). AI algorithms can also analyze public health data to predict areas with high stigma or low early diagnosis rates. Health authorities can then conduct targeted awareness campaigns and allocate resources more effectively (23).

By providing personal, confidential, and accessible support and information, AI empowers people to overcome their fears and misconceptions, ultimately improving health outcomes and reducing stigma.

## 8 Addressing accessibility challenges

AI and telemedicine enter into a symbiotic relationship. AI can improve telemedicine through advanced diagnostics, real-time monitoring, and personalized care. It automates administrative tasks, ensures data accuracy, and bridges language barriers, particularly useful in regions with limited access to healthcare (20, 23–26).

**Figure 2:**
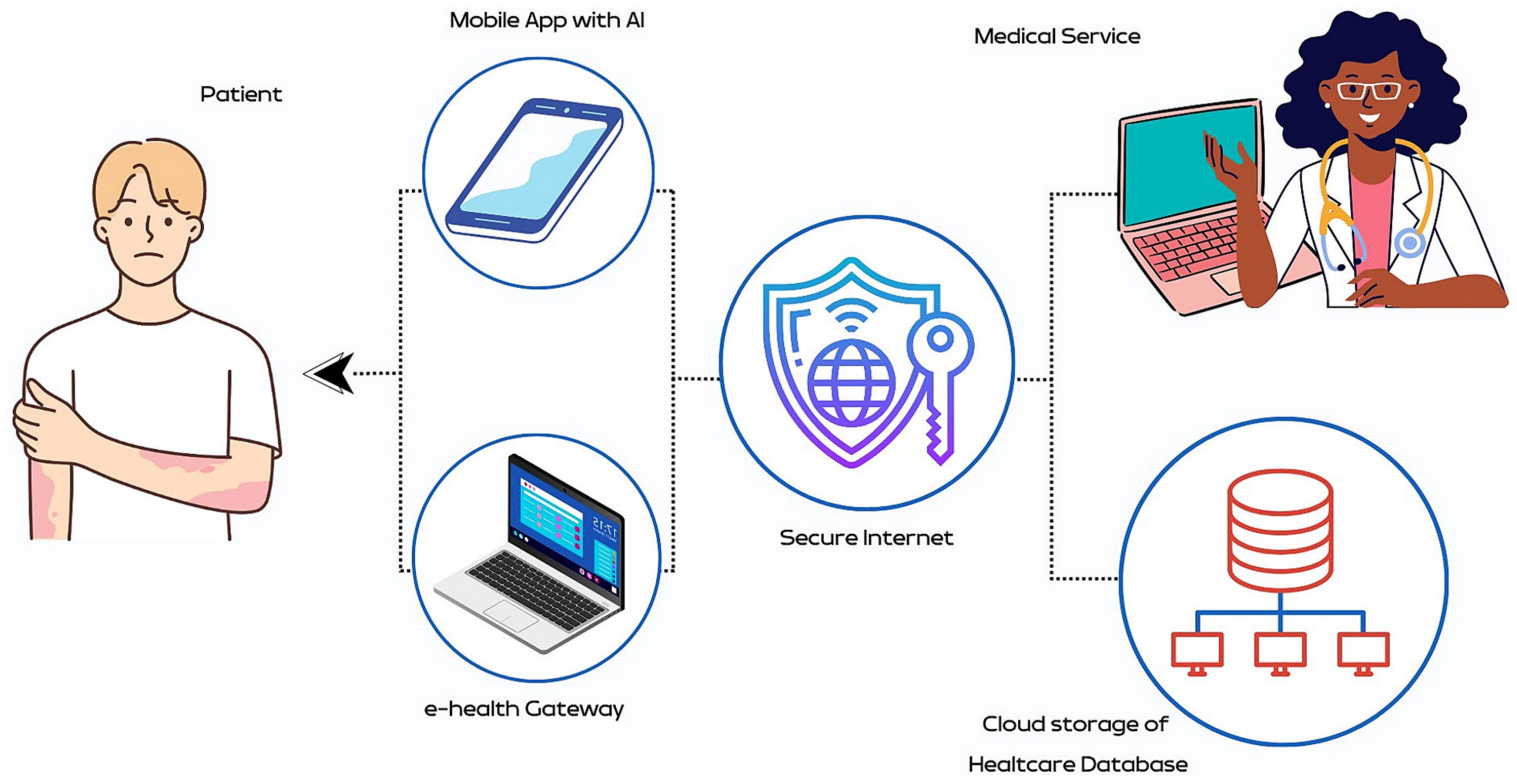
Artificial intelligence (AI)-enhanced telemedicine for remote monitoring of Hansen’s disease (HD). It features an AI-powered mobile app that tracks symptoms and provides diagnostic guidance, enhancing patient engagement and self-care. The e-health gateway securely integrates patient data into healthcare networks via the internet, ensuring data privacy. Healthcare professionals use telemedicine platforms to access and manage this data, supported by a secure cloud database for real-time analysis. This integrated system ensures personalized and timely medical services, improving care continuity and health outcomes for HD patients, even in remote areas.

In teleconsultations, AI can support healthcare providers with cutting-edge diagnoses. For example, AI algorithms analyze medical images in real time (13–15). AI-driven devices continuously monitor vital signs and health data, which are transmitted to healthcare professionals to track progress in real time and adjust treatment plans (Figure 2) (24, 27, 28). Importantly, AI is constantly learning and adapting as more patient data becomes available, refining recommendations over time. Chatbots and virtual assistants are streamlining telemedicine by taking over administrative tasks, appointment scheduling, medical reminders, and language translation, improving communication between patients and doctors (20). Additionally, AI can be used in patient triage to prioritize critical cases for immediate assessment. Moreover, AI collects and analyzes data for medical research, clinical trials, and telemedicine best practices (16).

This synergy of AI and telemedicine has great potential to make healthcare accessible, efficient, and effective, regardless of geographical restrictions.

## 9 Ethical considerations and data privacy

The integration of AI into HD diagnosis and treatment offers immense potential but requires ethical diligence. The sensitive nature of patient data requires reliable data protection and security. Patients must be able to trust that their data is protected from unauthorized access. Therefore, healthcare providers and AI developers have a responsibility to implement robust safeguards to ensure data privacy (29).

Informed consent is fundamental to ethical medical practice, ensuring patients comprehensively understand the role of AI in their care, encompassing its benefits, limitations, and potential risks. Patients also have the legitimate right to comprehend how AI systems arrive at diagnosis and treatment recommendations. Ethical AI operates with respect for patients’ autonomy, recognizing their authority in shaping their treatment plans. Developers are responsible for prioritizing transparency and offering clear explanations and justifications for AI-driven decisions. While AI provides valuable recommendations, the ultimate authority in healthcare decisions rests with the patient. AI should function as a tool that empowers patients and amplifies their choices rather than attempting to undermine them (30).

Another critical ethical dimension relates to bias and fairness. AI algorithms, especially machine learning models, learn from historical data. If the training data used to develop these models is biased, the AI system may inherit and perpetuate this bias. In the context of HD, AI systems can also be influenced by biases originating from patient demographics, access to healthcare, or previous diagnostic decisions. Biased AI can exacerbate inequalities in healthcare by potentially misdiagnosing or undertreating certain groups. Several strategies are needed to reduce bias in AI diagnosis, such as diversifying training data, deploying debiasing techniques, and conducting regular audits to ensure fairness and accuracy (16, 30).

Ethical principles demand that AI not replace medical professionals but support them. It is crucial not only for patient safety but also for ensuring medical ethics that physicians retain control over AI-driven decisions. Integrating AI into healthcare raises complex accountability issues, mainly when errors and adverse outcomes occur. To address these concerns and protect patients’ rights and trust in AI-enabled healthcare, it is essential to establish clear responsibilities and liability issues between healthcare providers, AI developers, and healthcare institutions (29).

Emphasizing transparency, fairness, patient autonomy, and continuous improvement ensures the ethical use of AI technologies that benefit patients while respecting their rights and dignity throughout their healthcare journey.

## 10 Necessary steps to build a trustworthy AI-based approach for HD services

Specific guidelines for validating AI algorithms are needed for the diverse use of AI in healthcare. This ensures the rigorous testing and reliability of AI algorithms in HD management. To create a trustworthy and reliable AI-based approach to HD programs and services, several key steps must be followed. These steps include data collection, pre-processing, feature selection, algorithm development, training, and validation of AI models, all guided by ethical considerations and collaboration with healthcare experts. The ultimate goal is to develop AI systems compliant with regulatory requirements and enable more effective disease management and improved support for people affected by HD (29).

## 11 Conclusion

The future of AI in the management of neglected tropical diseases, particularly HD, holds immense promise for equitable and accessible healthcare. Pioneering initiatives have already made significant progress in the application of AI in public health, providing insight into its transformative potential in the diagnosis and treatment of HD. Collaboration between healthcare organizations, technology companies, and research institutions will be critical to achieving this potential.

While resource allocation for AI initially seemed simple, its integration into current medical settings is complex. Financial issues are less of a concern now, as many healthcare facilities can afford AI technology. However, two critical factors are proving to be the most significant obstacles: the availability of high-quality data and the adaptability of existing processes to AI models. Questions arise about the seamless integration of data into AI models. Can data flow effectively into the models? Is there an automatic mechanism for merging data from different sources? It is also important to consider the location and usability of the AI models. Even advanced AI solutions might be underutilized in practice without usability, underscoring the need to address these questions thoroughly.

## Data availability statement

The original contributions presented in the study are included in the article/supplementary material, further inquiries can be directed to the corresponding author/s.

## Author contributions

PD: Conceptualization, Data curation, Investigation, Project administration, Writing – original draft, Writing – review & editing. RY: Writing – review & editing. BF: Data curation, Investigation, Writing – review & editing. BO: Data curation, Investigation, Writing – review & editing. SL: Writing – review & editing. RL: Writing – review & editing, Conceptualization, Data curation, Investigation, Writing – original draft.

## References

[1] Deps P Collin SM de Andrade VLG. Hansen’s disease case detection in Brazil: a backlog of undiagnosed cases due to COVID-19 pandemic. J Eur Acad Dermatol Venereol. (2022) 36:e754–5. doi: 10.1111/jdv.18307, PMID: 35680545 PMC9347646

[2] World Health Organization, Organisation mondiale de la Santé. Relevé Épidémiologique Hebd. Wkly Epidemiol Rec. (2021) 96:421–44.

[3] Weekly Epidemiological Record. (2023), [EN/FR] -World: ReliefWeb. Available at: https://reliefweb.int/report/world/weekly-epidemiological-record-wer-8-september-2023-vol-98-no-37-pp-409-430-enfr

[4] Deps P Collin SM. Mycobacterium lepromatosis as a second agent of Hansen’s disease. Front Microbiol. (2021) 12:698588. doi: 10.3389/fmicb.2021.698588, PMID: 34566911 PMC8461103

[5] White C Franco-Paredes C. Leprosy in the 21st century. Clin Microbiol Rev. (2015) 28:80–94. doi: 10.1128/CMR.00079-13, PMID: 25567223 PMC4284303

[6] Alemu Belachew W Naafs B. Position statement: LEPROSY: diagnosis, treatment and follow-up. J Eur Acad Dermatol Venereol. (2019) 33:1205–13. doi: 10.1111/jdv.15569, PMID: 30945360

[7] Control of Neglected Tropical Diseases, SEARO Regional Office for the South East Asia (RGO) (2023). Towards zero leprosy: Global leprosy (Hansen’s Disease) strategy 2021–2030. Available at: https://www.who.int/publications-detail-redirect/9789290228509

[8] World Organization of Health. (2023). Control of Neglected Tropical Diseases, SEARO Regional Office for the South East Asia (RGO). Interruption of Transmission and Elimination of Leprosy Disease. World Health Organization. Regional Office for South-East Asia. Available at: https://www.who.int/publications-detail-redirect/9789290210467

[9] Deps PD Nasser S Guerra P Simon M Birshner RDC Rodrigues LC. Adverse effects from multi-drug therapy in leprosy: a Brazilian study. Lepr Rev. (2007) 78:216–22. doi: 10.47276/lr.78.3.216, PMID: 18035772

[10] Smith WC van Brakel W Gillis T Saunderson P Richardus JH. The missing millions: a threat to the elimination of leprosy. PLoS Negl Trop Dis. (2015) 9:e0003658. doi: 10.1371/journal.pntd.0003658, PMID: 25905706 PMC4408099

[11] Warne G Mukhier M. Application of digital technologies to leprosy programmes. Lepr Rev. (2021) 92:182–5. doi: 10.47276/lr.92.2.182

[12] Yang J Li X Sun Y Zhang L Jin G Li G. Global epidemiology of leprosy from 2010 to 2020: a systematic review and meta-analysis of the proportion of sex, type, grade 2 deformity and age. Pathog Glob Health. 116:467–76. doi: 10.1080/20477724.2022.2057722 PMC9639561 35510339

[13] Barbieri RR Xu Y Setian L Souza-Santos PT Trivedi A Cristofono J. Reimagining leprosy elimination with AI analysis of a combination of skin lesion images with demographic and clinical data. Lancet Reg Health Am. (2022) 9:100192. doi: 10.1016/j.lana.2022.100192, PMID: 36776278 PMC9903738

[14] Yotsu RR Ding Z Hamm J Blanton RE. Deep learning for AI-based diagnosis of skin-related neglected tropical diseases: a pilot study. PLoS Negl Trop Dis. (2023) 17:e0011230. doi: 10.1371/journal.pntd.0011230, PMID: 37578966 PMC10449179

[15] Beesetty R Reddy SA Modali S. et al, Leprosy skin lesion detection: an AI approach using few shot learning in a small clinical dataset. Indian J Lepr. (2023) 95:89–102.

[16] Pai VV Pai RB. Artificial intelligence in dermatology and healthcare: an overview. Indian J Dermatol Venereol Leprol. (2021) 87:457–67. doi: 10.25259/IJDVL_518_19, PMID: 34114421

[17] de Andrade Rodrigues RS Heise EFJ Hartmann LF Rocha GE Olandoski M de Araújo Stefani MM. Prediction of the occurrence of leprosy reactions based on Bayesian networks. Front Med. (2023) 10:1233220. doi: 10.3389/fmed.2023.1233220, PMID: 37564037 PMC10411956

[18] Portelli S Myung Y Furnham N Vedithi SC Pires DEV Ascher DB. Prediction of rifampicin resistance beyond the RRDR using structure-based machine learning approaches. Sci Rep. (2020) 10:18120. doi: 10.1038/s41598-020-74648-y, PMID: 33093532 PMC7581776

[19] Deps P Delboni L TIA O Collin SM Andrade MA ELN M. Steps towards eliminating Hansen’s disease stigma. Int Health. (2023) 15:iii7–9. doi: 10.1093/inthealth/ihad050, PMID: 38118154 PMC10732667

[20] Xu L Sanders L Li K Chow JCL. Chatbot for health care and oncology applications using artificial intelligence and machine learning: systematic review. JMIR Cancer. (2021) 7:e27850. doi: 10.2196/27850, PMID: 34847056 PMC8669585

[21] van der Schyff EL Ridout B Amon KL Forsyth R Campbell AJ. Providing self-led mental health support through an artificial intelligence-powered chat bot (Leora) to meet the demand of mental health care. J Med Internet Res. (2023) 25:e46448. doi: 10.2196/46448, PMID: 37335608 PMC10337342

[22] Babu NV Kanaga EGM. Sentiment analysis in social media data for depression detection using artificial intelligence: a review. SN Comput Sci. (2022) 3:74. doi: 10.1007/s42979-021-00958-1, PMID: 34816124 PMC8603338

[23] De Souza MLM Lopes GA Branco AC Fairley JK Fraga LADO. Leprosy screening based on artificial intelligence: development of a cross-platform app. JMIR Mhealth Uhealth. (2021) 9:e23718. doi: 10.2196/23718, PMID: 33825685 PMC8060869

[24] Latif S Qadir J Farooq S Imran M. How 5G wireless (and concomitant technologies) will revolutionize healthcare? Future Internet. (2017) 9:93. doi: 10.3390/fi9040093

[25] Dahoklory DF Haryanto J Indarwati R. The application of digital health as a nursing solution for leprosy patients during the COVID-19 pandemic: a systematic review. JPMA J Pak Med Assoc. (2023) 73:S170. doi: 10.47391/JPMA.Ind-S2-38 37096727

[26] Saif-Ur-Rahman KM Islam MS Alaboson J Ola O Hasan I Islam N. Artificial intelligence and digital health in improving primary health care service delivery in LMICs: a systematic review. J Evid-Based Med. (2023) 16:303–20. doi: 10.1111/jebm.12547, PMID: 37691394

[27] Dananjayan S Raj GM. 5G in healthcare: how fast will be the transformation? Ir J Med Sci. (2021) 190:497–501. doi: 10.1007/s11845-020-02329-w 32737688

[28] Devi DH Duraisamy K Armghan A Alsharari M Aliqab K Sorathiya V. 5G Technology in healthcare and wearable devices: a review. Sensors. (2023) 23:2519. doi: 10.3390/s23052519, PMID: 36904721 PMC10007389

[29] Geis JR Brady AP Wu CC Spencer J Ranschaert E Jaremko JL. Ethics of artificial intelligence in radiology: summary of the joint European and north American multisociety statement. J Am Coll Radiol. (2019) 16:1516–21. doi: 10.1016/j.jacr.2019.07.028, PMID: 31585696

[30] Keskinbora KH. Medical ethics considerations on artificial intelligence. J Clin Neurosci. (2019) 64:277–82. doi: 10.1016/j.jocn.2019.03.001 30878282

